# Strategies for diversity: medical clowns in dementia care - an ethnographic study

**DOI:** 10.1186/s12877-016-0325-z

**Published:** 2016-08-18

**Authors:** Margareta Rämgård, Elisabeth Carlson, Elisabeth Mangrio

**Affiliations:** Department of Care Science, Malmö University, Jan Waldenströms gata 25, 205 06 Malmö, Sweden

## Abstract

**Background:**

As nursing homes become increasingly diverse, dementia care needs a wider range of culturally responsive strategies for individual and collective social interactions. While previous studies conclude that medical clowns have positive effects on verbal and non verbal social interactions, research is lacking from the perspective of residents' cultural background. The aim of this study was to identify interaction strategies employed by medical clowns in culturally diverse dementia care settings.

**Method:**

An ethnographic approach was used and data were collected through observation of interactions between medical clowns and residents with dementia in two nursing homes during a ten week period.

**Results:**

The observations showed that the medical clowns interacted with residents by being tuned in and attentive to the residents as individuals with a unique life-history, confirming each person´s sense of self. The clowns used sensory triggers, encouragement and confirmation in culturally responsive ways to bond socially with the residents in their personal spaces. The clowns involved objects in the daily environment that were meaningful for the residents, and paid attention to significant places and habits in the past. The clowns further contributed to joint interaction in the common spaces in the nursing homes, using music and drama.

**Conclusion:**

The strategies employed by medical clowns in activities with older people with dementia appear to support social interaction. The medical clowns used the social and material environment in culturally responsive ways to strengthen individuals’ sense of self, while contributing to a sense of togetherness and interaction among residents in the common spaces. Findings suggest that both verbal and non-verbal cultural content affected social interaction. The non-demanding encouraging way the clowns tuned in to the residents as individuals could help nurses and staff members improve ways of communication in social activities inside the nursing home.

## Background

With increasing mobility, nursing home environments are becoming more diverse with respect to the background of both staff and residents, and the proportion of foreign-born older people is growing. 1 To provide adequate care, additional research on intercultural aspects in gerontology is called for, exploring new approaches to interaction and communication in culturally diverse nursing settings. The strategies employed by medical clowns appeared to be a potentially interesting area of study, since they are trained to use body, emotions and culture in their interaction.

The present study is an ethnographic study of interaction between residents with dementia and medical clowns visiting two nursing homes during a ten-week period. The aim of the study was to identify strategies for interaction employed by medical clowns in activities with older people with dementia within the context of the nursing homes where they live. To obtain data on interaction with residents of different backgrounds, the selection of nursing homes comprised a home with mostly Swedish-born residents, and a home with many foreign-born residents. We are not aware of other studies on interactions between medical clowns and people with dementia in nursing homes that devotes attention to residents born in other countries.

Medical clowns have become a more frequent activity in nursing homes around the world [1], and their use of both verbal and non-verbal interaction makes medical clowns of particular interest for dementia care. Identifying strategies that support positive interaction with people with dementia additionally has potential implications for nursing practice, since certain strategies could be used in other activities, or applied as an integrated part of daily care. While this study discusses the care of foreign-born residents, the observed strategies have applications more generally for developing social interaction in nursing home settings.

### The medical clown as a cultural activity

A considerable body of research about older people with dementia living in nursing homes has established the need for stimulation through social and cultural activities [2–4]. Single studies [3, 5] indicate that nurses need to break the daily routines and find stimulating activities for people with dementia in nursing homes. Stimulating cultural activities may involve music, dance, or even drama [6]. Activities in dementia care have also been found to affect mood and behaviour. A systematic review by Testad et al. [7] found good evidence supporting the value of personalised pleasant activities, such as reminiscence therapy and music, for the treatment of agitation and to improve mood. Several studies also discuss cultural activities as useful tools for communication and social interaction in dementia care [8–10]. The effects of music and dance may go beyond reducing behavioural and psychological symptoms, since music and dance are so closed linked to life histories and personal identities [11–13].

Medical clowns have been studied both as instances of cultural activities, and from the angle of so-called ‘humour therapy’. Medical clowns have been working with hospitalised children for more than two decades, in Sweden as well as in other countries [14–16]. Evidence from studies over a broad range of methodologies, including randomised control studies, as well as qualitative observation or interview studies, converges concerning beneficial effects. Medical clowns reduce anxiety among children in hospitals undergoing medical procedures, for example venous puncture, examination for sexual abuse and preoperative care [17–21]. Barkmann et al. [22] argue that the concurrence of results on this point is sufficiently robust to justify evidence-based practice using medical clowns.

Some researchers within the field, [20] argue that medical clowns have developed techniques using a relational approach, which supports empowerment processes. It has been suggested that this approach helps children to manage anxiety and loneliness [16].

Benefits have also been observed for the care environment as a whole, including parents and hospital staff [16, 23]. Projects in the USA illustrate how observing medical clowns’ work can increase caregivers’ sensitivity to the atmosphere created around children in hospitals [24]. Parents believed that their children were happier after the clown visits, and thought it was beneficial for the overall atmosphere in the hospitals [22, 25]. Research thus substantiates that the presence of medical clowns promotes well-being in health care settings for children. Warren & Spitzer [1] conclude that medical clowns strike a balance between being entertainers and health workers. They contribute to reducing stress for patients and supporting families.

### Medical clowns and people with dementia

A number of studies on older people with dementia living in nursing homes concern the effects of humour therapy and quality of life [26]. For example, it was found that agitation levels decreased significantly in nursing homes where medical clowns worked with humour therapy, compared to homes where no cultural activities took place [27]. Findings in studies by Raviv [28] and Hendricks [29] confirm that medical clowns had a beneficial and effective impact when working with persons with dementia, integrating skills such as drama, music and dance. Humour can stimulate social interactions in dementia care [30]. Positive emotions and laughter may enable people with dementia to cope better with their illness, improve immune defence, increase pain tolerance and decrease stress response. Such results point to effects that resemble intervention studies involving psychosocial interventions [7] where social activities reduce aggressive and depressive behaviour among older people with dementia.

Research on medical clowns in dementia care shows especially positive effects on psychosocial function, cognition and duration of agitation [1, 27] A few recent studies on medical clowns in nursing homes look more closely at interaction between clowns and older people with dementia using ethnographic methods and observations [26, 29]. These studies suggest that the relationship between staff and residents is improved, and that the presence of medical clowns helps staff to support the feelings and skills of the residents Results also indicate that medical clowns can support empowerment processes among older people with dementia, using the beneficial effects of elements in the environment on interaction. Using the body is significant for the results [29]. By working with music, dance and movement, the clown is able to strengthen the older people’s cultural sense of self and their corporeality.

## Method

The study adopted a focused ethnographic approach, enabling in-depth understanding of particular behaviours and processes in a smaller group of people in a distinct setting [31, 32]. Focused ethnography is especially useful when the focus of a study is limited to a discrete community [33]. Observation allows the researcher to collect data on the context from a first-hand perspective, adding the extra dimension of the influence of the physical environment [34]. In addition, observations can help us to comprehend situations involving a vulnerable population, who may have difficulties in expressing their views verbally [26]. For older people with dementia, the ability to communicate may be reduced by diseases and conditions that also affect facial expression, body language and non-verbal communication more generally [26].

### Setting

In 2013, the project “Clown Rounds” was initiated by the Regional Board of Cultural Activities in four municipalities in southern Sweden. The purpose of the project was to develop new cultural activities for people with dementia, living in nursing homes run by the municipalities.

Two nursing homes that were part of the “Clown Rounds” were chosen for the present study through convenience sampling [35]. One nursing home in an urban area had residents originating from several countries, while the other was located in a rural area, and residents had all been born in Sweden. The nursing homes had individual rooms, which the residents had furnished themselves, as well as common spaces with details intended to remind residents of what was in fashion when they were younger. Each nursing home had approximately 30 residents. At each site, the sessions were set up by two medical clowns working together and who returned to the same home for the duration of the project. The clowns had professional backgrounds as musicians and actors, with an additional training within dementia care. They wore costumes that is usually recognised as clown attributes, for example shoes that are too large, baggy trousers, colourful shirts, and the red nose. Before they started their activity, the nursing staff gave the medical clowns and the researcher an in-depth life story related to every person with dementia living in the nursing home (Table 1). The clowns were active both in shared spaces and the individual rooms within the nursing home.

**Table 1:** Social and medical characteristics of the 60 residents

Age Range	Gender	Diagnosis	Ethnicity	Mobility
60-95 years	33 women	Dementia 60	Sweden 40	Bedridden 7
	27 men		Finland 4	Wheelchair 15
			SouthEurope 10	Walk assist 30
			(Bosnia, Greece and Spain)	Walking alone 8
			Asia 6	
			(Afghanistan, Iraq and Iran)	

### Procedure

The clown visits took place once a week at the two nursing homes during a ten week long period during autumn 2013 and spring 2014. Each weekly session lasted for three to four hours. The total length of observation was 35 h. The last author conducted all the observations, taking the role as an overt observer. Overt observation means that the participants know they are being observed, but the observer do not actively participate in any situation that might occur [36]. She visited the nursing homes during the sessions when the two medical clowns were active, following the clowns in their work and taking notes.

During the observations, the nursing staff were present but not engaged in the interactions. On one occasion, two family members were visiting their relative during the clown session, and were thus part of the observation. Observations were unstructured. No checklist was used, but observations were focused specifically towards interaction between clowns and the older persons [37]. Notes were taken directly, and reflections were added by the observer immediately after each session [34]. The last author (EM) is a novice observer; however, authors (EC and MR) are experienced observers and qualitative researchers and the research team met continuously during the time of data collection to discuss and reflect the progress of observations.

### Data analysis

Following the ethnographic approach, data collection and analysis is a simultaneous process [31]. After each observed clown session, field notes were transcribed to a neat copy to guide coming observations and as a way to organize data in order to uncover patterns of interaction. The transcribed texts were read and reread several times to gain a general sense of data and ensure a deep understanding. Data were then coded and compared so categories could develop, using the constant comparative method were each item of data was compared to other data categorised in the same way, allowing for new categories and finally the formation of the overarching theme [31] (Table 2). Field notes and transcribed interviews as well as categories and codes that emerged during analysis were checked and discussed until agreement by the three authors to ensure credibility [38].

**Table 2:** Some examples illustrating the analysis process

Meaning unit	Code	Category	Theme
*What a handsome guy you have on the photo! He was my husband, replies Karin. He was not so old, she continues. Was he also from Scania (south of Sweden)? Yes, she replies, and proceeds to explain that she had an earlier marriage as well. Was he also from Scania? No, she replies. He was from Värmland*	Social interaction starts by giving her attention, pointing out that she has a handsome guy on a photo in her apartment. The person with dementia then continues the conversation and answers properly	Encouragement and confirmation	Being tuned in and attentive
*The medical clowns admired Elsa’s fine fabrics hanging on the wall, and asked if she made these? Yes, she replied. I made them when I worked the night-shift in the hospital. You have been working a lot of night-shifts then, the medical clowns asked. . Elsa responded: Yes, I have. The clowns continued by asking: How do you sleep at night now? As a child, Elsa responded with a smile adding: It is great to see new faces.*	Social interaction starts by giving attention and admiring fabrics hanging on the wall in her apartment, asking if she made it herself. The person with dementia then continues the conversation, answers properly and smiles	Encouragement and confirmation	Being tuned in and attentive
*The medical clowns picked up a silk fabric with sequins on and tied it on Nina’s hips and the medical clowns started to play and dance. Nina started to laugh loudly but looked a little bit embarrassed, then laughed again. Now the other medical clown took the silk fabric around his hips and started to dance and shaking the hips. Nina who was sitting at the table next to her daughters then looked away and said something to her daughters and then laughed again. The daughters said with amazement that Nina formulated a whole sentence in her own language, which she hadn´t done for a long time. (Field note, Oct. 16th)*	Social interactions starts by given attention to a silk fabric. The clown then imitating a belly dancer from the Middle East, The person with dementia responded by laughing and continue to interact with the clown in her language of origin- her next of kind said that she had not spoken for a very long time	Using a sensory trigger	Being tuned in and attentive

## Results

The findings illustrate the strategies deployed by the medical clowns. The overarching theme that emerged was ‘Being tuned in and attentive’, which were subdivided into the categories: Using sensory triggers; Encouragement and confirmation in the interaction; Paying attention to places in the past; and Use of music as a joining activity in shared spaces.

### Being tuned in and attentive

The analysis of the observations showed how the medical clowns were tuned in and attentive in a culturally responsive way in all contacts with the residents in the nursing homes. They were attentive to individual needs on a personal level, so they could start building a relationship. Relational strategies employed by the medical clowns in the interaction involved sensory triggers, encouragement and confirmation. Participants responded both verbally and physically, displaying a variety of emotions. The clowns payed attention to places in the past that were significant for the individual in the one-on-one interaction, as well as contributing to interaction among the residents in spaces of joint activities in the nursing home.

### Using sensory triggers

When visiting the residents in their rooms, the medical clowns used different kinds of accessories related to the senses to initiate and support interaction, such as a red nose, a soft toy in the shape of a pig, a shawl and silk fabric. Mostly, the residents were sitting in a chair looking at the door, but some residents were resting on their beds. When they entered the room, the clowns used sensory cues as an invitation to interact. For example, the clowns might point at their red noses, the resident would look at the nose, and this could serve to initiate interaction. In order to connect with residents with a Middle Eastern background, the clowns draped a shawl around their hips to mimic a belly dancer, singing and dancing. Such strategies are exemplified by the following field note:

*The medical clowns picked up a silk fabric with sequins on and tied it on Nina’s hips and the medical clowns started to play and dance. Nina started to laugh loudly but looked a little bit embarrassed, then laughed again. Now the other medical clown took the silk fabric around his hips and started to dance and shaking the hips. Nina who was sitting at the table next to her daughters then looked away and said something to her daughters and then laughed again. The daughters said with amazement that Nina formulated a whole sentence in her own language, which she hadn´t done for a long time. (Field note, Oct. 16th)*

In order to reach an Arabic-speaking person, the medical clowns sang Arabic songs, which the resident responded to by smiling and expressing appreciation through facial expressions. With residents lying in their beds, the medical clowns used physical contact to initiate interaction by stroking their cheek, and residents responded by smiling. The silk fabric was used as a sensory cue that the residents could touch with their hands. When the person expressed contentment at feeling the soft fabric, the medical clowns could proceed to interact in other ways.

Several interaction sequences thus typically started by stimulating the senses, such as touch, sight and hearing, and using sensory cues in an individualised manner.

### Employing encouragement and confirmation in the interaction

Words of encouragement and confirmation were frequently used in the clowns’ interaction with residents in their own rooms in the nursing home. The clowns talked about the residents’ appearance in a positive manner, for instance saying that they looked beautiful. The clowns also offered recognition and showed interest in the person as an individual by asking for their name, and then connecting it to something positive. They put small stickers with stars on the residents’ chests saying: “You are such a fantastic person”. The clowns tried their best to understand and kept the dialogue going even when talking to one of the residents who could only mumble and was not able to speak clearly. They commented on and discussed the flowers and furniture in the residents’ rooms, and in response to such comments, the residents started to interact socially with the clowns, giving proper answers and smiling. An example of personalised recognition and confirmation was when the medical clowns were pointing at a boat sculpture in one of the rooms. The resident reacted by suddenly starting to talk about fish, and the clowns then followed this line of thought, deepened the dialogue and expressed contentment and interest. Another example is when looking at a photograph of a resident’s husband, one of the clowns commented:

*What a handsome guy you have on the photo! He was my husband, replies Karin. He was not so old, she continues. Was he also from Scania (south of Sweden)? Yes, she replies, and proceeds to explain that she had an earlier marriage as well. Was he also from Scania? No, she replies. He was from Värmland (Midwestern Sweden) and she laughs. (Field Note, Nov. 13th)*

The medical clowns also encouraged the participants when saying goodbye, for instance by confirming how strong they were when shaking hands.

### Paying attention to places in the past

Another strategy used by the medical clowns was connecting to places earlier in the person’s life. One example was a blind person who had grown up in Italy:

*The medical clowns came to Francesco after knocking on his door and introduced themselves, and Francesco replied that he remembered them from the visit last week then he became quiet. The medical clowns then started to sing a song and Francesco then started to interact, he was smiling, laughing and mentioning that he remembered the song from Italy. Further on, Francesco told them that the song reminded him of the good old days in Italy and the specific Italian food and drinks. (Field Note, Oct, 9th)*

Residents born in Sweden also started interact better when the clowns paid attention to significant places in their past. When the medical clowns asked one of the residents sitting in a chair in her room if she came from a certain town in the north of Sweden, she answered yes. This question served as the start of a conversation about nature and the activities that used to take place in that part of the country, which then served as a focus for the interaction. She responded by smiling and looking content, reminded of this period in her life.

*The medical clown continues to talk with Karin about the mountains and Karin discusses this further and smiles contentedly. Karin says that there were many people who went skiing in the mountains, but she went sleigh-riding, and she shows how it was done with her arms. (Field Note, Oct 16th*)

It was generally observed that when the clowns started a conversation about places in their past, the residents remembered the places and that made them happy, supporting further interaction.

### Use of music as a joining activity in shared spaces

One strategy used by the medical clowns in the shared spaces inside the nursing home was to use different kinds of music, instruments and songs. Shared spaces included the larger dining rooms with tables and chairs, but there was also empty space for people with wheel chairs. During one of the sessions, when the clowns began to play for a person in those spaces, the other residents in the nursing home suddenly began stomping to the beat of the music, rolling the wheel chairs, swaying their feet and singing along with the music, and also started to dance with each other. The residents didn’t only respond to the clowns; many came up with suggestions of their own for songs they knew or started to sing by themselves. This space of joint activities was seen through the fellowship that was first created between the medical clowns and one of the residents, but was then spread to the others in the same space. The way activities with a particular resident could contribute to the general atmosphere is illustrated in the following field note.

*Bassem started drumming to the musical tune and the medical clowns came along playing instruments and dancing. Bassem laughed afterwards and everyone was applauding. The medical clown went up to another person, Nilufer, sitting in the same room. She tried to say something quietly and the medical clown asked if they should play a song and she nodded her head. When the song began, Roger, a fellow resident, joined in by moving his wheelchair back and forth. Soon after, Farhad swayed his feet, and Lisa, sang along with the song, while sitting and touching the silk fabric. When the medical clowns asked if they recognized the song, Jenny answered quietly “yes” and Roger answered loudly “yes”. Lisa who was still touching the silk fabric talked quietly with one of the medical clowns. The other medical clown continued to play, while several people continued to sing along with the song. Lisa, touching the silk fabric, took the initiative to sing another song and the medical clown started to play the song. (Field Note, Jan. 15th)*

This attention to each other was not only connected to the social interactions between the residents and the clowns generated by music, instruments and dance, but also related to rhythm and bodily movement in general. For example, when one of the medical clowns was stamping with her high heels while entering into the living room, this was followed by two residents, who started to sing along with each other. In all these activities, the residents started talking in a more interactive way to each other. Using music and dance, the clowns were able to awaken culturally specific associations for individual residents, at the same time that they seemed to create a sense of togetherness shared by all the residents in the common room.

In yet another situation, the medical clowns staged a fake wedding, involving the residents as audience and one person as a priest. The residents all showed signs of enjoying the show, smiling and talking to each other. The wedding game and the interactions during the performance were commented on by several people in the room.

These joint activities thus stimulated the social interactions among the residents in the whole nursing home. The medical clowns paid attention to everybody’s reactions and interacted in a way that maintained a friendly atmosphere and a sense of security during their visits.

## Discussion

Research on medical clowns suggests that humour therapy reduces agitation, mood and anxiety, while improving life quality [26–30]. Empowerment mechanisms seem to play a role in these results. Culture, environment and social interaction are all elements of such empowerment mechanism [20, 29]. The effects of humour therapy extend to other patients, family and staff [20, 26, 29]. Medical clowns thereby contribute not only to individual health and well-being, but to the environment as a whole [16]. This latter aspect clearly appears in the present study.

The observations showed that the medical clowns took care to ‘tune in’ to the person they were interacting with and be attentive to individual personalities and life histories. The clowns created an attachment and a social bond in the relationship by using sensory triggers, encouragement and confirmation and by paying attention to places and habits in the past in personalised one-on-one interaction. No withdrawals were observed from interactions in the residents’ own rooms, and the reason might be that the medical clowns were especially attentive not to insist for responses; interaction was grounded in sensitive and responsive playfulness.

Importantly, interaction was open-ended: it did not impose an agenda, nor was it intended to lead to a pre-established result. This could affect implications for clinical practice. The clowns could be highly respectful of each individual’s limits and personal interests because they were not obliged to accomplish specific results within a given time, while regular staff may not always be able to work with equal flexibility.

The observations showed reactions where residents responded positively, both verbally and physically, despite their cognitive problems and language barriers. This confirms findings from other research projects concluding that cultural activities are important for the well-being of people with dementia [2, 4, 7, 26]. Observations further showed that foreign-born residents suffering from dementia can actively participate in cultural activities even when verbal proficiency in the second language is reduced.

The clowns confirmed the person's own identity by relating the sensory cues to something in the past (such as specific music, dances and places), which resulted in improved social interaction.

This is line with Hendriks [29], which indicates the importance of activating older people’s cultural sense of self through body. By including a cultural dimension in the interaction, the clowns were thus able to establish a personalised relationship and strengthen the residents’ sense of self.

It appeared that the medical clowns stimulated the residents’ autobiographical memory by being attentive and interested when talking of places and activities in the past, This is significant, since it could decrease the risk of infantilisation [39].

More general research on autobiographical memory concludes that stimulating emotions by remembering our attachment to places in the past could strengthen our sense of self in the present [40, 41]. A problem in dementia care might therefore arise if caregivers and residents do not share sufficient cultural or geographical reference points to be able to work with reminiscence or give adequate responses and confirmations of self. With increased global mobility, caregivers will frequently have had geographical trajectories and cultural backgrounds that differ from the older people they take care of. Devoting attention to life-histories as well as learning more about the residents’ cultural background and geographical trajectories can increase opportunities for dialogue.

A considerable body of research suggests that staff should encourage identity support activities for people suffering from dementia [42–44]. By strengthening each individual's perception of themselves, cognitive ability could increase, which in turn affects their social interaction [5].

The medical clowns’ way of using objects in the environment as identity triggers seemed to strengthen the residents’ sense of self in a way that increased their well-being. They used specific sets of material objects, brought with them cultural artefacts associated to different places to form meaning based on earlier experience, and which helped to locate and bound reality conceptually. Since the present material world provides structure and continuity, as well as offering shared reference points in social interaction, objects and the physical environment could be used more consciously as a tool to strengthen people living with dementia.

Besides connecting to individual interests and life-histories, the medical clowns’ work also created social shared spaces inside the nursing home with a joyful atmosphere where the whole care unit interacted socially in a way that can be described as a system of interaction. Hyden [5] argues that joint activities are important for people living together in a place of care because they belong to an ecological system with a communicative ecosystem of collaboration, The medical clowns’ work resulted in collective collaborative processes between the clowns and the residents.

As nursing home environments become more diverse, the interaction strategies employed by medical clowns thus suggest a variety of approaches that can contribute to supporting intercultural dynamics among residents and improving interaction both for individual residents and in the environment as a whole.

### Trustworthiness

The transferability of this study could be limited since only two different nursing homes were represented in the study [38]. However the two nursing homes were located in different municipalities and residents had differing backgrounds, which could strengthen the credibility of the study. Credibility was further ensured through several steps. During the analysis process, all data and emerging categories were checked and discussed until agreement was reached by all authors. Representative excerpts from field notes are used to illustrate the findings in the data (see Table 2 below). The role of the researcher needed to be taken into consideration and reflected upon to ensure dependability [38]. For this reason, reflective notes about the researcher’s role were regularly written during and after field sessions.

This procedure also enabled reflections and discussions among all three authors concerning reflexivity. The question of reflexivity [45] has been addressed and discussed among the three authors based on the extensive field-notes that were made for each observation as a means to illuminate the interaction between the researcher and the studied group [34]. It is important to raise the question of reactivity as the presence of the researcher as observer may have influenced the behaviour of the studied group with the risk of altering the interaction between the medical clowns and the person with dementia. The author who collected data through observations emphasised throughout the data collection period that she was not an employee of the two nursing homes, and therefore could not participate in any nursing tasks or resident care. None of the researchers had any previous experience from the studied nursing homes nor where they acquainted to the health care staff, the older people or the medical clowns participating in the study. All three authors are experienced qualitative researchers with a background as registered nurses, but without clinical experience from dementia care.

## Conclusion

The medical clowns appeared to increase social interaction for people with dementia in the nursing homes investigated in this study. The way medical clowns tuned in and were attentive in a cultural responsive way to the present as well as the past, connecting to both the social and the material environment, could strengthen the residents’ sense of self. Working in personalised and culturally responsive ways with different senses increased residents’ possibilities to interact with their bodies as well as in words. The study supports conclusions from earlier research suggesting that medical clowns contribute to strengthening social interactions within nursing homes for persons with dementia, adding a geographical and cultural dimension. Medical clowns confirm the sense of self for persons with dementia, using encouragement, culturally anchored cues for interaction and by connecting to significant places in the past. Strategies that contribute to increased responsiveness and social interaction include sensory triggers, using meaningful objects in the present environment, shared activities, involving body, music and drama. The presented strategies appeared potentially useful for the staff to support the cognitive abilities of people with dementia. Cultural background and geographical trajectories of nursing home residents need to be considered by stakeholders responsible for dementia care. More research is also needed to further evaluate the contributions of medical clowns for older people with dementia who are foreign-born or who spent their youth in other countries.

## Abbreviations

MR: Margareta Rämgård
EC: Elisabeth Carlson
EM: Elisabeth Mangrio

## Footnotes

1 In Sweden today 234 889 people in the age group 65 + are foreign-born (12, 2 %), and the proportion of foreign born older people in need of nursing care is expected to grow from 12, 2 % 2014 – 16 % by 2020 (Statistics Sweden, 2015).
